# Elovl6 regulates mechanical damage-induced keratinocyte death and skin inflammation

**DOI:** 10.1038/s41419-018-1226-1

**Published:** 2018-12-05

**Authors:** Yoshiyuki Nakamura, Takashi Matsuzaka, Satoko Tahara-Hanaoka, Kazuko Shibuya, Hitoshi Shimano, Chigusa Nakahashi-Oda, Akira Shibuya

**Affiliations:** 1 Department of Immunology, University of Tsukuba, 1-1-1 Tennodai, Tsukuba, Ibaraki 305-8575 Japan; 2 Department of Internal Medicine (Endocrinology and Metabolism), Faculty of Medicine, University of Tsukuba, 1-1-1 Tennodai, Tsukuba, Ibaraki 305-8575 Japan; 3 Life Science Center for Survival Dynamics, Tsukuba Advanced Research Alliance (TARA), University of Tsukuba, 1-1-1 Tennodai, Tsukuba, Ibaraki 305-8575 Japan; 4 International Institute for Integrative Sleep Medicine (WPI-IIIS), University of Tsukuba, 1-1-1 Tennodai, Tsukuba, Ibaraki 305-8575 Japan; 5 AMED-CREST, Japan Agency for Medical Research and Development (AMED), 1-7-1, Ohte-machi, Chiyoda-ku, Tokyo, 100-0004 Japan

## Abstract

Mechanical damage on the skin not only affects barrier function but also induces various immune responses, which trigger or exacerbate skin inflammation. However, how mechanical damage-induced skin inflammation is regulated remains incompletely understood. Here, we show that keratinocytes express the long-chain fatty-acid elongase Elovl6. Mice deficient in Elovl6 showed higher levels of cis-vaccenic acid (CVA) in the epidermis and severe skin inflammation induced by mechanical damage due to tape stripping than did wild-type mice. CVA accelerated tape stripping-triggered keratinocyte death and release of danger-associated molecular patterns (DAMPs) such as high-mobility group box 1 protein (HMGB-1) and IL-1α, which induced production of proinflammatory cytokines and chemokines IL-1β and CXCL-1 by keratinocytes. Our results demonstrate that Elovl6 regulates mechanical damage—triggered keratinocyte death and the subsequent dermatitis.

## Introduction

The mechanical damage induced by physical forces—including stretching, compression, and friction—on epithelial and endothelial cells plays a critical role in tissue homeostasis^1,2^. The mechanical damage not only affects the barrier function of the skin but also induces various immune responses^3^, which trigger inflammation at the site of the stress on the skin. Moreover, mechanical damage on the skin exacerbates the inflammation in patients with inflammatory skin diseases. For example, scratching of itching lesions exacerbates the skin inflammation in atopic dermatitis (AD), which is called the itch-scratch cycle^3^. In addition, scratching induces development of new skin lesions in psoriasis, well-known as the Koebner phenomenon^4^. However, how mechanical damage-induced skin inflammation is regulated remains incompletely understood.

Elongation of long-chain fatty acids family member 6 (Elovl6) is a rate-limiting microsomal enzyme that catalyzes the elongation of saturated and monounsaturated fatty acids^5^. Elovl6 elongates palmitate (PA) (C16:0) to stearate (SA) (C18:0) and palmitoleate (POA) (C16:1n-7) to cis-vaccenic acid (CVA) (C18:1n-7)^5^. Elovl6 is highly expressed in the white adipose tissue and liver^6^. Elovl6 is involved in metabolic diseases, such as insulin resistance^7^ and atherogenesis^5^, as well as inflammatory diseases, including attenuated high-fat-diet-induced hepatic inflammation^8^ and regulated bleomycin-induced pulmonary fibrosis^9^. In addition, Elovl6 is highly expressed in the skin^6^, which is one of the most lipid-enriched organs. Lipids in the skin play crucial roles in homeostasis; they are involved in epidermal permeability and barrier function^10^, the composition of microbiota^11^, epithelialization^12^, and inflammation^13^. In the current study, we examined whether long-chain fatty-acid composition regulated by Elovl6 is involved in mechanical damage-induced skin inflammation.

## Results

### *Elovl6*^*−/−*^ mice show exacerbated mechanical damage-induced skin inflammation

**Fig. 1:**
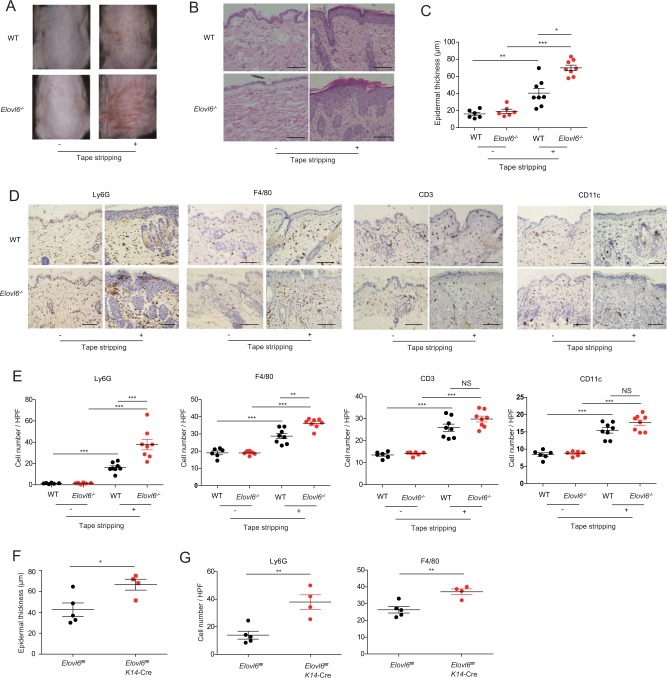
*Elovl6*^−/−^ mice show exacerbation of dermatitis. **a**–**e** Representative gross findings (**a**), histology (hematoxylin and eosin staining) (**b**), epidermal thickness (**c**), immunohistochemistry, and cellular quantification of infiltrated neutrophils (Ly6G), macrophages (F4/80), T cells (CD3), and dendritic cells (CD11c) in the dorsal skin of wild-type (WT) (*n* = 6 or 8) and *Elovl6*^*−/−*^ (*n* = 6 or 8) mice before and on day 9 after the start of tape stripping (**d**, **e**). **f**, **g** Epidermal thickness (**f**) and numbers of infiltrated neutrophils (Ly6G), and macrophages (F4/80) in the skin (**g**) of *Elovl6*^*fl/fl*^ (*n* = 5) and *Elovl6*^fl/fl^*K14*-Cre (*n* = 4) on day 9 after the start of tape stripping. Black bars indicate scale (50 μm) (**b**, **d**). Error bars indicate 1 SD; *, *P* < 0.05; **, *P* *<* 0.01, ***, *P* *<* 0.001. NS not significant, HPF high-power (400 × ) field. Data are representative of three (**a–e**) and two (**f**, **g**) independent experiments

Tape stripping, which mimics scratching, is a well-established method for inducing mechanical stress or damage on the skin^14^. To investigate the role of Elovl6 in mechanical damage-induced skin inflammation, we established a mouse model of dermatitis by using repeated tape stripping. After this treatment, erythema was more severe in *Elovl6*^*−/−*^ mice than in wild-type mice (Fig. 1a). Moreover, the epidermis was thicker, and neutrophil and macrophage infiltrations were significantly greater, as analyzed by immunohistochemical studies, in *Elovl6*^*−/−*^ mice than in wild-type mice. In contrast, T cells and dendritic cells were comparable between two genotypes of mice (Fig. 1b–e). Since *Elovl6* expression was higher in the epidermis than in the dermis (Supplementary Fig. S1A), we speculated that Elovl6 is expressed in keratinocytes. Indeed, the epidermis in mice deficient in Elovl6 specifically in the keratinocytes (*Elovl6*^fl/fl^ *K14*-Cre mice) showed significantly decreased Elovl6 expression (Supplementary Fig. S1B). As in *Elovl6*^*−/−*^ mice, *Elovl6*^fl/fl^ *K14*-Cre mice also showed increased epidermal thickness and neutrophil and macrophage infiltrations after tape stripping compared with control mice (Fig. 1f, g) (Supplementary Fig. S1C).

**Fig. 2:**
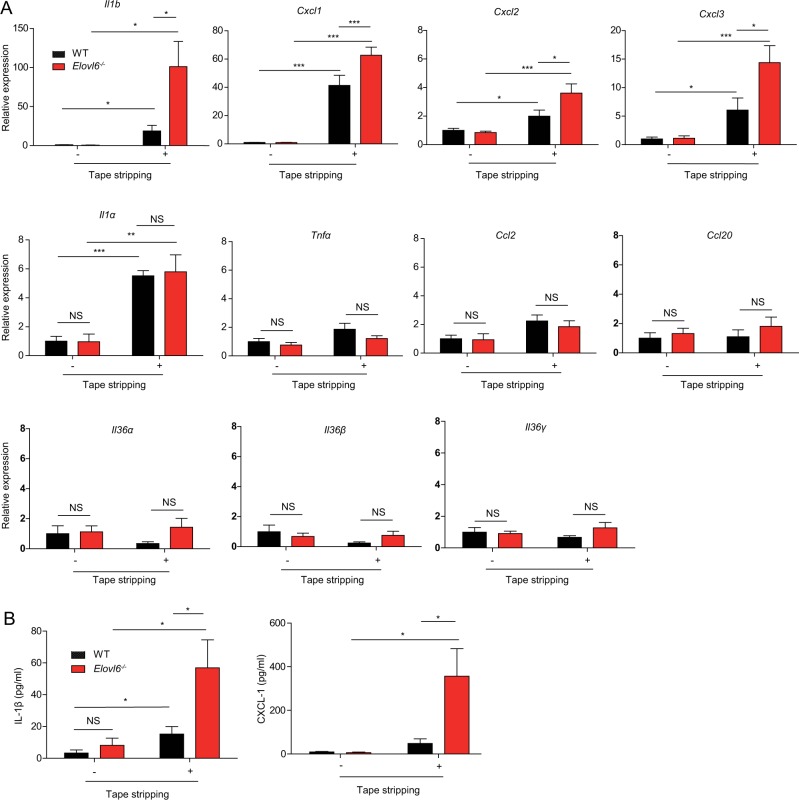
Elovl6 suppresses mechanical damage-induced IL-1β, CXCL-1, CXCL-2, and CXCL-3 production. **a** Quantitative RT-PCR analysis for expressions of cytokines and chemokines in epidermis of wild-type and *Elovl6*^*−/−*^ mice isolated before and 6 h after tape stripping (*n* = 10 in each group). **b** Epidermis was isolated before, and 12 h after, tape stripping from wild-type and *Elovl6*^*−/−*^ mice and cultured for 24 h. The concentrations of IL-1β and CXCL-1 in the supernatants were measured by using cytometric bead array (*n* = 11 per group). Error bars indicate 1 SD; *P < 0.05; **P *<* 0.01, ***P *<* 0.001. NS not significant. Data are representative of two independent experiments

To investigate how Elovl6 suppressed mechanical damage-induced skin inflammation, we examined the expression levels of pro-inflammatory and anti-inflammatory cytokines and chemokines, which were reported to be potentially involved in dermatitis^15^. Among them, transcript levels of *Il1a, Il1b*, *Cxcl1*, *Cxc12*, and *Cxcl3* in the epidermis were increased in both wild-type and *Elovl6*^*−/−*^ mice after tape stripping (Fig. 2a). Moreover, *Elovl6*^*−/−*^ mice showed higher expression of *Il1b*, *Cxcl1*, *Cxc12,* and *Cxcl3* than did wild-type mice after tape stripping (Fig. 2a). In accordance with these results, the concentrations of IL-1β and CXCL-1 were significantly higher in the culture supernatants of *Elovl6*^*−/−*^ epidermis harvested from mice after tape stripping than in those from the wild-type epidermis (Fig. 2b). Together, these results suggest that Elovl6 suppresses mechanical damage-induced skin inflammation.

### *Elovl6*^*−/−*^ mice show increased keratinocyte death after mechanical damage

**Fig. 3:**
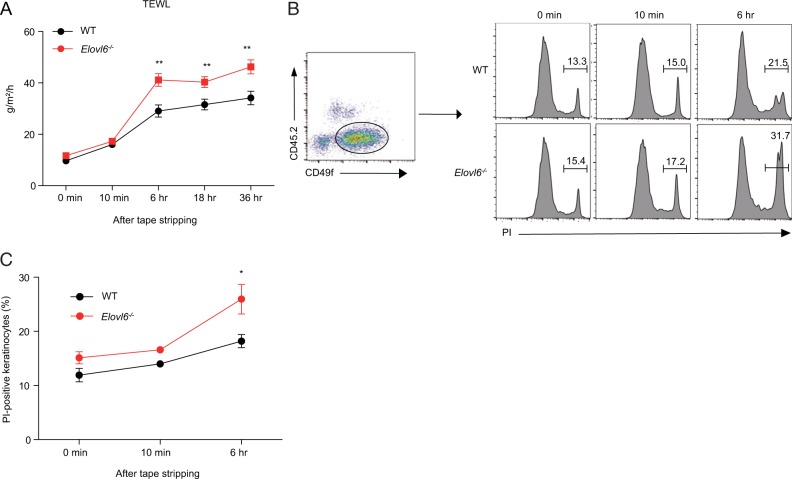
Elovl6 suppressed mechanical damage-induced keratinocyte death. **a** Transepidermal water loss (TEWL) of 6–8-week-old wild-type and *Elovl6*^*−/−*^ mice was measured before and after tape stripping (*n* = 14). **b**, **c** Flow cytometry of epidermal cells isolated from the skin of wild-type and *Elovl6*^*−/−*^ mice at the indicated time after tape stripping. Epidermal cells were stained with anti-CD45.2, anti-CD49f, and propidium iodide (PI), and the proportion of PI^+^ cells in CD45.2^*−*^CD49f^+^ keratinocytes was calculated (*n* = 6–10 per group). Error bars indicate 1 SD; *P *<* 0.05; **P *<* 0.01. Data are representative of two independent experiments

Since fatty acids play an important role in skin barrier function^10^, we next examined whether Elovl6 deficiency affects barrier function by the transepidermal water loss (TEWL) test. Although it was comparable between naive wild-type and *Elovl6*^*−/−*^ mice, *Elovl6*^*−/−*^ mice showed increased TEWL than did wild-type mice 6 h or later after tape stripping (Fig. 3a). We found that the proportion of dead keratinocytes (CD45.2^−^ CD49f^+^ cells), as determined by propidium iodide (PI) staining by using flow cytometry, were increased in *Elovl6*^*−/−*^ mice more than in wild-type mice 6 h after tape stripping (Fig. 3b, c), suggesting that the increased keratinocyte death led to the barrier dysfunction in *Elovl6*^*−/−*^ mice. Together, these results suggest that Elovl6 suppressed mechanical damage-induced keratinocyte death and skin inflammation.

### CVA induces keratinocyte death

**Fig. 4:**
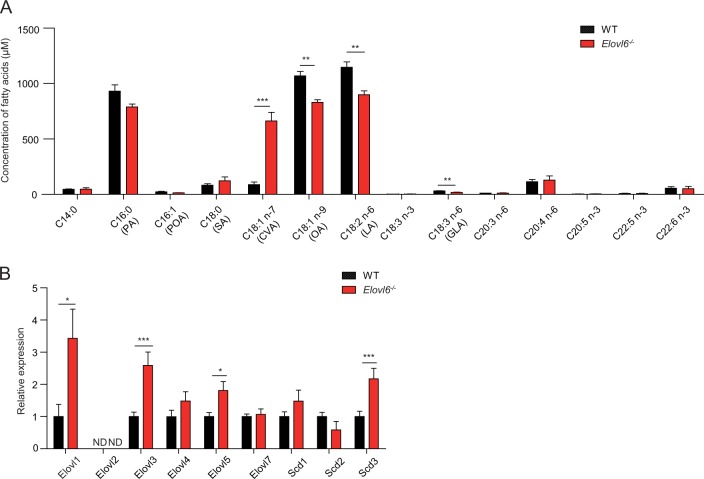
Elovl6 suppressed cis-vaccenic acid (CVA) production in keratinocytes. **a** Fatty acid composition of epidermis in wild-type and *Elovl6*^*−/−*^ mice (*n* = 4 per group). **b** Quantitative RT-PCR analysis of the epidermis for the expression of elongations of long-chain fatty acids (*Elovl1*~*7*) and stearoyl-CoA desaturases (*Scd1*~*3*) in wild-type and *Elovl6*^*−/−*^ mice (*n* = 10). Error bars indicate SD. *P < 0.05, **P < 0.01, ***P < 0.001. ND not detected, PA palmitate, POA palmitoleate, SA stearate, CVA cis-vaccenic acid, OA oleic acid, LA linoleic acid, GLA γ-linolenic acid. Data are representative of two independent experiments

We then analyzed the fatty acid composition of the epidermis of wild-type and *Elovl6*^*−/−*^ mice. *Elovl6*^*−/−*^ mice had increased CVA levels and decreased OA, linoleic acid (LA; C18:2n-6), and γ-linoleic acids (GLA; C18:3n-6) than did wild-type mice (Fig. 4a). *Elovl6*^*−/−*^ mice also showed greater epidermal expression of the long-chain fatty acid elongases *Elovl1*, *Elovl3*, and *Elovl5* and of the stearoyl-CoA desaturase *Scd3* than did wild-type mice (Fig. 4b). Among these, *Elovl5* and *Scd3* may influence CVA generation through the elongation of POA (C16:1n-7)^16^ and by the conversion of PA to POA^17^, respectively (Supplementary Fig. S3).

**Fig. 5:**
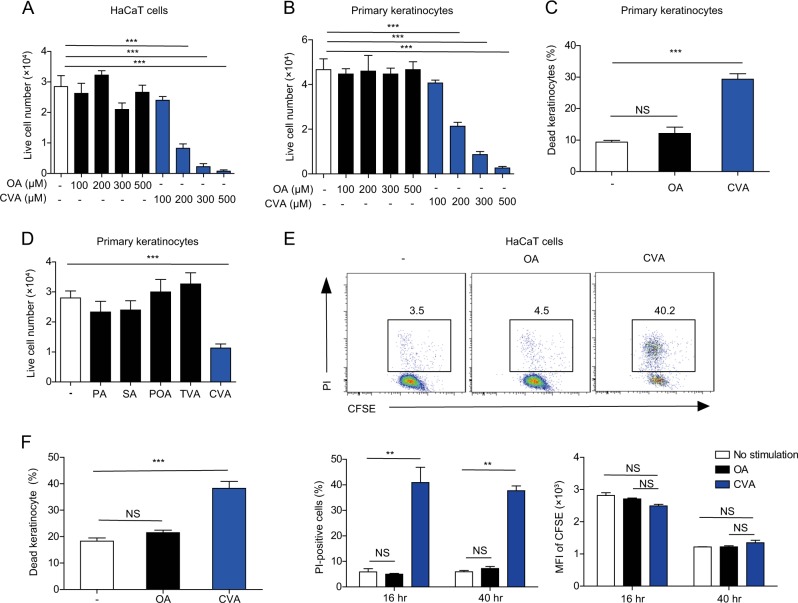
CVA induces keratinocyte death in the skin. **a**, **b** Number of live cells in cultures of HaCaT cells (**a**) and primary keratinocytes isolated from the skin of new born mice (**b**) 16 h after stimulation with indicated concentration of OA or CVA was calculated using trypan blue exclusion test (*n* = 3 per group). **c** Primary keratinocytes were stimulated for 6 h with 300 μM of OA or CVA, stained with propidium iodide (PI) and analyzed by flow cytometry for the proportion of PI-positive (i.e., dead) cells (*n* = 4 or 5 per group). **d** Number of live cells in primary keratinocytes 16 h after stimulation with 300 μM of PA, SA, POA, TVA, or CVA was calculated using trypan blue exclusion test (*n* = 3 per group). **e** HaCaT cells were labeled with CFSE and stimulated or not with 300 μM of OA or CVA for 16 or 40 h. Cells were then stained with PI and analyzed by flow cytometry. **f** Epidermal cells of mice that received topical application of ethanol (control) or 45 mM of OA or CVA to the dorsal skin for 6 h were stained with anti-CD45.2, CD49f, and PI and analyzed by flow cytometry for PI-positive (i.e., dead) CD45.2^*−*^CD49f^+^ keratinocytes. Error bars indicate SD. **P < 0.01, ***P < 0.001. NS not significant, OA, oleic acid, CVA cis-vaccenic acid, PA palmitate, SA stearate, POA palmitoleate, TVA trans-vaccenic acid. Data are representative of more than two independent experiments

Since CVA showed the most prominent change in *Elovl6*^*−/−*^ mice compared with wild-type mice, we speculated that CVA might be involved in keratinocyte death. To address this hypothesis, a human keratinocyte cell line HaCaT or primary keratinocytes derived from mice were cultured in the presence of CVA. We found that CVA decreased the numbers of live cells of HaCaT cells and primary keratinocytes in a dose-dependent manner (Fig. 5a, b) and increased the proportion of dead primary keratinocytes (Fig. 5c). In contrast, neither oleic acid (OA), PA, POA, SA, nor trans-vaccenic acid (TVA) influenced the number of live primary keratinocytes after culture (Fig. 5d). In addition, CVA decreased the number of live peritoneal macrophages as well (Supplementary Fig. S4A). CVA did not affect the proliferation of HaCaT cells but instead increased the number of dead cells compared with those after the addition of OA (Fig. 5e), thus indicating that treatment with CVA-induced cell death of HaCaT cells. This cell death was not affected by triacsin C, an inhibitor of long-chain acyl-CoA synthetases^18^ (Supplementary Fig. S4B), suggesting that CVA itself, but not its metabolites, induced death of HaCaT cells. Morphologic analyses under transmission electronic microscopy demonstrated increased plasma membrane rupture without blebbing in the keratinocytes after CVA treatment (Supplementary Fig. S4C). In vivo, we found that topical application of CVA, but not OA, at a dose of 45 mM to the dorsal skin of wild-type mice increased the proportion of dead keratinocytes (Fig. 5f). Anti-cleaved caspase-9 (CC9) antibody did not stain CVA-treated dead keratinocytes (Supplementary Fig. S4D). Together, these results suggest that CVA induced non-apoptotic cell death. Pretreatment with necrostatin-1 or necrosulfonamide, which are inhibitors of receptor-interacting protein 1 (RIP1) kinase and mixed lineage kinase domain-like protein (MLKL), respectively, did not suppress the CVA-induced death of keratinocytes (Supplementary Fig. S4E), suggesting that the cell death due to CVA likely was not necroptosis^19^. These combined results suggest that CVA induced necrosis rather than programmed cell death of keratinocytes. In addition, treatment with inhibitors of oxidative stress (IM-54) or cyclophilin D (cyclosporine A) did not influence the cell death (Supplementary Fig. S4E), suggesting that the CVA-induced necrosis of keratinocytes was independent of oxidative stress (IM-54) or cyclophilin D-mediated changes in mitochondrial permeability^20^.

### CVA increased IL-1β, CXCL-1, CXCL-2, and CXCL3 production

**Fig. 6:**
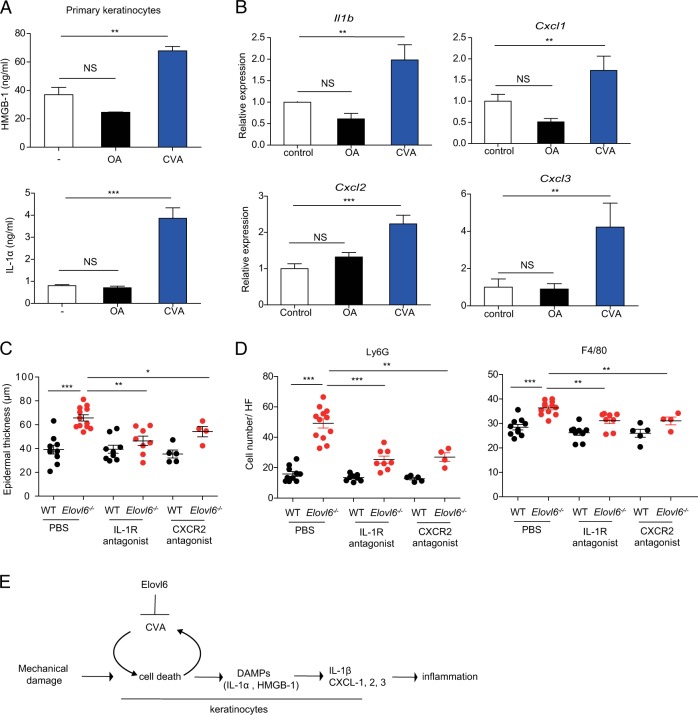
CVA increased IL-1β, CXCL-1, CXCL-2, and CXCL-3 production. **a** Enzyme-linked immunosorbent assay of HMGB-1 (*n* = 4 per group) and cytokine bead array of IL-1α (*n* = 3 per group) in the supernatant of cultured primary keratinocytes 10 h after initiation of stimulation with 300 μM of OA or CVA. **b** Quantitative RT-PCR analysis of *Il1β*, *Cxcl1*, *Cxcl2,* and *Cxcl3* in the epidermis of wild-type mice 6 h after topical application of ethanol (control) (n = 10) or 15 mM of OA (n = 13) or CVA (*n* = 14). **c**, **d** Wild-type and *Elovl6*^*−/−*^ mice received intradermal and intraperitoneal administration of PBS (*n* = 10 and 12, respectively), an IL-1 receptor antagonist (*n* = 9 and 8, respectively), or a CXCR-2 antagonist (*n* = 5 and 4, respectively) daily for 9 days, from the beginning on the day of tape stripping. The skin was then analyzed for epidermal thickness by histology (hematoxylin and eosin staining) (**c**) and the number of neutrophils (Ly6G) and macrophages (F4/80) by immunohistochemical studies (**d**) on day 9. **e** A proposed signal pathway from mechanical damage onto the skin to inflammation. Error bars indicate SD; *P *<* 0.05; **P *<* 0.01, ***P *<* 0.001; NS not significant, OA oleic acid, CVA cis-vaccenic acid. Data are representative of more than two independent experiments

Since CVA induced non-apoptotic cell death, we then examined whether CVA increased the release of DAMPs from dead cells. The addition of CVA, but not OA, to cultures of primary keratinocytes increased the concentrations of HMGB-1 and IL-1α in the supernatants (Fig. 6a). Furthermore, stimulation of primary keratinocytes derived from wild type or *Elovl6*^*−/−*^ mice in vitro and of the epidermis from the either genotype of mice in vivo with HMGB-1 or IL-1α induced *Il1β*, *Cxcl1*, *Cxcl2, and Cxcl3*, and the expression levels of these cytokine transcripts did not differ between both genotypes of mice (Supplementary Fig. S5A, B). These results suggest that CVA enhanced IL-1β, CXCL-1, CXCL-2, and CXCL-3 production by keratinocytes via HMGB-1 or IL-1α. Indeed, we found that topical application of CVA, but not OA, at a dose of 45 mM to the dorsal skin increased the expressions of *Il1β*, *Cxcl1*, *Cxcl2, and Cxcl3* in the epidermis (Fig. 6b). Finally, treatment with either antagonist of IL-1 receptor or CXCR-2 intradermally and intraperitoneally reduced epidermal thickness and the number of neutrophils and macrophages in the skin of *Elovl6*^*−/−*^ mice (Fig. 6c, d) (Supplementary Fig. S5C). Taken all together, these results suggest that tape stripping triggered keratinocyte death and release of HMGB-1 and IL-1α, which then stimulated the surrounding live keratinocytes to produce IL-1β, CXCL-1, CXCL-2, and CXCL-3, thus exacerbating skin inflammation with acanthosis and infiltration of neutrophils and macrophages. Elovl6 deficiency accelerated tape stripping-triggered keratinocyte death, which possibly not only caused the skin barrier dysfunction but also increased the DAMPs release from the dead keratinocytes, thus exacerbating dermatitis (Fig. 6e).

## Discussion

Previous reports demonstrated that *Elovl6*^*−/−*^ mice had increased PA level and decreased OA level in the lung and liver of *Elovl6*^*−/−*^ mice compared with wild-type mice^7,9^. Consistently, we found that OA level was also decreased in the epidermis. Unlike the previous reports, however, PA was not increased in the epidermis. Instead, *Elovl6*^*−/−*^ mice had increased CVA levels and decreased linoleic acid (LA; C18:2n-6) and γ-linoleic acids (GLA; C18:3n-6) in the epidermis. Although the biologic function of CVA has been poorly understood, we showed that CVA induced cell death of keratinocytes triggered by mechanical damage. It remains unclear whether the aberrant composition of fatty acids other than CVA is also involved in keratinocyte death in *Elovl6*^*−/−*^ mice.

Previous studies have demonstrated that, compared with OA and SA, both CVA and TVA (dose, 30 μM) significantly suppress the growth of HT-29 tumor cells after culture for 9 days^21^. Moreover, CVA leads to greater hydrolysis of phosphoinositides in the plasma membrane than does TVA^21^. In the present study, we showed that CVA at concentrations of 200 μM or greater induced the death of primary keratinocytes, thus suggesting that 200 μM is the minimal dose required to damage the plasma membrane sufficiently to induce necrosis in vitro. In addition, given that TVA did not induce keratinocyte death, the cytotoxic effect of CVA may be structure-dependent. Whereas trans-unsaturated fatty acids have a linear structure and can be packed regularly in the plasma membrane, cis-unsaturated fatty acids, such as OA and CVA, which have a characteristic angular kink, may distort the structure of the lipid bilayer and thus destabilize the plasma membrane^22^. Therefore, we speculate that incorporation of CVA into the plasma membrane creates a bulky three-dimensional structure compared with those associated with other cis-monounsaturated fatty acids and thus induces cell death by disrupting the plasma membrane. Further studies are required to determine the detailed mechanism of CVA-induced cell death.

Fatty acids reportedly play important roles in modulating the severity of dermatitis in mouse models. For example, a high-fat diet enriched with oleic acid impairs contact hypersensitivity responses to trinitrochlorobenzene and fluorescein isothiocyanate^23^. In addition, oral administration of docosahexaenoic acid leads to the generation of regulatory T cells, which thus attenuate dinitrochlorobenzene-induced dermatitis^24^. Moreover, topical or oral application of linoleic acid and TVA, which are enriched in milk fat, decreases the severity of ovalbumin-induced dermatitis^25^.

AD is one of the most common skin diseases with Th2-dominant immune responses and is characterized by pruritic and eczematous skin lesions. Mechanical damage, such as scratching, increases the severity of AD by diminishing the epidermal barrier function and production of pro-inflammatory cytokines^3,26^. The lipids in the stratum corneum, the outermost layer of the epidermis, are crucial for the barrier function of the skin. The amount of ceramides with very long-chain fatty acids is decreased in the stratum corneum in AD patients^27^ and the chain length of fatty acids of ceramide is negatively correlated with impaired barrier function in AD patients^28^. Consistently, *Elovl1*^*−/−*^ and *Elovl4*^*−/−*^ mice, both of which demonstrate a global decrease in ceramides with very long-chain fatty acids in the stratum corneum, show impaired barrier function^29,30^.

The lipids are also involved in the aberrant immune response in AD patients. Previous reports demonstrated increased arachidonic acid and its bioactive lipid-mediator metabolites, such as prostaglandin D2 (PGD2) and leukotriene B4 (LB4) in the AD lesion^31–33^. Whereas PGD2 induced Th2 cell recruitment^34^, LB4 promoted activation and recruitment of inflammatory cells, including neutrophils, eosinophils, monocytes/macrophages, and T cells^35^. Recently, Berdyshev E. et al. showed that Th2-related cytokines IL-4 and IL-13 downregulated Elovl6 expression in human keratinocytes, and Elovl6 expression was indeed downregulated in the lesion of AD^36^.

On the other hand, psoriasis is characterized by well-demarcated scaly erythema and plaque, whose lesions show hyperproliferation of keratinocytes and neutrophil infiltration^37^. The skin lesions of psoriasis are triggered or exacerbated by mechanical damage, which is well-known as the Koebner phenomenon^4^. Abnormal fat metabolism is also an important factor in the pathogenesis of psoriasis^38^. In addition, alterations in epidermal lipids, such as increased phospholipids, triacylglycerols, and cholesterol and decreased phosphatidylinositol and phosphatidylserine, are observed in the epidermis of psoriasis patients^39^. Our study revealed that dysregulated fatty acid composition by Elovl6 deficiency in keratinocytes accelerated tape stripping-triggered keratinocyte death, which exacerbated skin inflammation, thus suggesting that Elovl6 may play important roles in the pathogenesis of AD and psoriasis through suppressing mechanical damage-induced skin inflammation.

## Experimental procedures

### Mice

*Elovl6*^*−/−*^ mice on the C57BL/6J background were described previously^7^. C57BL/6J mice raised under specific pathogen-free conditions were purchased from Clea Japan (Tokyo, Japan). *K14*-Cre on the B57BL/6 background were purchased from Jackson Laboratories (Bar Harbor, ME, USA). *Elovl6*^*fl/fl*^ mice were crossed with *K14*-Cre transgenic mice to generate Elovl6-knockout mice specifically in keratinocytes (*Elovl6*^*fl/fl*^ *K14*-Cre). Mice between 8 and 10 weeks of age were used for the experiments. All experiments were performed in accordance with the guidelines of the animal ethics committee of the University of Tsukuba Animal Research Center.

### Tape stripping

Mechanical damage-induced dermatitis was generated, as previously described^40^. In brief, a 2.5 × 2.5-cm area of the dorsal skin was shaved and tape-stripped 20 times by using adhesive tape (Johnson and Johnson); a 1 × 1-cm piece of sterile gauze moistened with 100 μl of PBS was placed on the shaved skin and secured with transparent bio-occlusive tape (Tegaderm Roll, 3 M, Maplewood, Minnesota, USA) to prevent the mice from licking the area. These procedures were repeated every other day until analysis.

### Cytokine measurement of epidermis or cultured keratinocytes

Dorsal skin samples before and after tape stripping were resected from adult mice and incubated in RPMI medium in the presence of dispase II (3 mg/ml) (Wako Pure Chemical, Osaka, Japan) for 1 h at 37 °C under 5% CO_2_. The epidermis was then separated from the dermis under a stereomicroscope. Samples of the epidermis (diameter, 4 mm) were cultured in 50 μl of DMEM containing 10% FBS in a 96-well plate at 37 °C under 5% CO_2_ for 24 h and the concentrations of IL-1β, CXCL-1, TNFα, and IL-10 in the culture supernatants were measured by using cytometric bead arrays (CBA) (BD Biosciences) according to the manufacturer’s protocol. The skin of newborn mice (younger than 3 days old) was incubated in CnT-07 medium (CELLnTEC Advanced Cell Systems) in the presence of dispase II (1 mg/ml) (CELLnTEC Advanced Cell Systems) at 4 °C for 16 h. The epidermis was isolated from the skin and incubated in Accutase (CellnTEC Advanced Cell Systems) at room temperature for 20 min. Keratinocytes collected were maintained in CnT-07 medium (CELLnTEC Advanced Cell Systems) according to the manufacturer’s protocol, and then stimulated with long-chain fatty acids at 37 °C under 5% CO_2_ for 10 h. The culture supernatants were analyzed for IL-1α and HMBG-1 by flow cytometry using cytokine beads array (CBA) and for HMGB-1 using an ELISA KIT II (Shino-test Corporation). Keratinocytes were also stimulated with IL-1α or HMGB-1 at 37 °C under 5% CO_2_ for 3 h and analyzed for *Il1b* and *Cxcl1* by quantitative real-time PCR analysis (qRT-PCR).

### Histology

For histologic analysis, mouse skin was fixed in 10% formalin, embedded in paraffin, sectioned, and stained with hematoxylin and eosin. For immunohistochemical analyses, paraffin-embedded sections were deparaffinized in xylene and rehydrated before antigen retrieval by boiling in citrate buffer (0.01 M citrate containing 0.5% Tween 20, pH 6.0). The sections were incubated in 10% bovine serum albumin in PBS at room temperature for 1 h and then stained with rat anti-Ly6G antibody (RB6-8C5, 1:250 dilution; Abcam), rabbit anti-F4/80 antibody (D2S9R, 1:100 dilution; Cell Signaling Technology), rabbit anti-CD3 antibody (SP7, 1:500 dilution; Abcam), or rabbit anti-CD11c antibody (N418, 1:100 dilution; Cell Signaling Technology) overnight at 4 °C, followed by biotinylated anti-rat IgG antibody or anti-rabbit IgG antibody (1:500; Vector Laboratories) and Vectastatin ABC reagent (Vector Laboratories) at room temperature for 60 mins and 30 mins, respectively. Finally, the sections were stained with DAB Peroxidase Substrate Kit before imaging (Vector Laboratories). For analyses of epidermal thickness or cell number, 18 randomly selected sites were evaluated by using microscopy (MZ-X710, Keyence, Osaka, Japan) and its associated software.

### Cell death and proliferation analyses

Primary keratinocytes from neonatal epidermis, prepared as described above, or human HaCaT cells were maintained in CnT-07 medium, as described above, and DMEM with 10% FBS, respectively, at 37 °C under 5% CO_2_. Peritoneal macrophages were harvested by lavage of the peritoneal cavity and suspended in DMEM containing 10% FBS. Primary keratinocytes, HaCaT cells, and peritoneal macrophages were seeded onto 48-well plates at a density of 1 × 10^5^ cells and 2 × 10^5^ cells/well, respectively. After incubation at 37 °C for 2 h, the cells were washed with PBS three times to remove unattached cells and stimulated with different concentrations of free fatty acids dissolved in ethanol (final dose of 100–500 μM), including oleic acid (OA), palmitic acid (PA), stearic acid (SA), palmitoleic acid (POA) (Wako Pure Chemical), trans-vaccenic acid (TVA), or cis-vaccenic acid (CVA) (Sigma-Aldrich).

The number of live cells was counted using 0.4% trypan blue (Thermo Fisher Scientific). For proliferation assay, these cells were stained or not with 10 μM of CFSE (Invitrogen) for 5 min at 37 °C before fatty acid stimulation, according to the manufacturer's instructions. Cells were then stained with propidium iodine (PI) and analyzed for PI-positive and PI-negative cell populations and CFSE dilution by flow cytometry. For the analysis of cell death in vivo, skin tissue was incubated in 0.5% trypsin (Wako) in PBS, and separated into epidermis. Epidermal cells were stained with CD45.2 (clone:104, BD Pharmingen), CD49f (clone: GoH3, Miltenyi Biotec), and PI, and then analyzed by flow cytometry.

### Cytokine stimulation

Primary mouse keratinocytes were stimulated with bovine HMGB-1 (Chondrex, Redmond, Washington, USA) or mouse IL-1α (Miltenyi Biotec, Bergisch Gladbach, Germany). For stimulation of keratinocytes in vitro, primary keratinocytes were stimulated with 500 ng/ml of bovine HMGB-1 or mouse IL-1α. For stimulation of keratinocytes in vivo, 200 ng of bovine HMGB-1 or mouse IL-1α in 50 μl of PBS was injected intradermally.

### Fatty acid composition

Lipids from the mouse epidermis were extracted by using the method of Bligh and Dyer^41^. In brief, epidermis was extracted with chloroform/methanol (1:2, v/v) solution. One molar NaCl solution and chloroform were added to break the monophase and incubated on ice for 10 min. After centrifugation at 300 G for 5 min, aqueous solution was discarded and the phase of chloroform was evaporated using nitrogen gas. Following the addition of acetonitrile/6 N HCl (90/10, v/v), samples were incubated at 100 °C for 45 min. Finally, liquid–liquid extraction^42^ with ethyl acetate was performed and the reconstituted samples were injected into an optimized LC/MS/MS system. The relative abundance of each fatty acid was quantified by gas chromatography.

### Transepidermal water loss (TEWL) test

TEWL was measured on the dorsal skin of wild-type and *Elovl6*^*−/−*^ mice between 8 and 10 weeks of age by Tewameter^®^ TM 300 (Integral) before and after tape stripping^30^. Measurements were performed in triplicate for each mouse.

### Antagonist treatment

To neutralize the IL-1 receptor, mice received 200 μl of the IL-1 receptor antagonist anakinra (10 mg/ml) (Kineret, Swedish Orphan Biovitrum) intradermally and 300 μl of the same concentration of the antagonist intraperitoneally daily during the induction of dermatitis by the mechanical stress or OVA treatment. To block CXCR-2, mice received 200 μl of a CXCR-2 antagonist (0.25 mg/ml in PBS containing 1% DMSO) (SB225002, Cayman Chemical, Ann Arbor, Michigan, USA) intradermally and 150 μg of the same antagonist (0.5 mg/ml in PBS containing 1% DMSO) intraperitoneally daily during the induction of dermatitis by the mechanical stress.

### Statistical analyses

Statistical analyses were performed by using an unpaired, two-tailed Student’s *t* test (GraphPad Prism 6, GraphPad Software, La Jolla, USA). A *P* value less than 0.05 was considered to be statistically significant.

## Supplementary information

Supplementary figures

supplementary figure legends

supplementary experimental procedures
