# Supplementary figures and images for: Elovl6 regulates mechanical damage-induced keratinocyte death and skin inflammation
Source: Cell Death Dis. 2018 Dec 5;9(12):1181. doi: 10.1038/s41419-018-1226-1 (PMC6281680; doi:10.1038/s41419-018-1226-1)

Figure S1

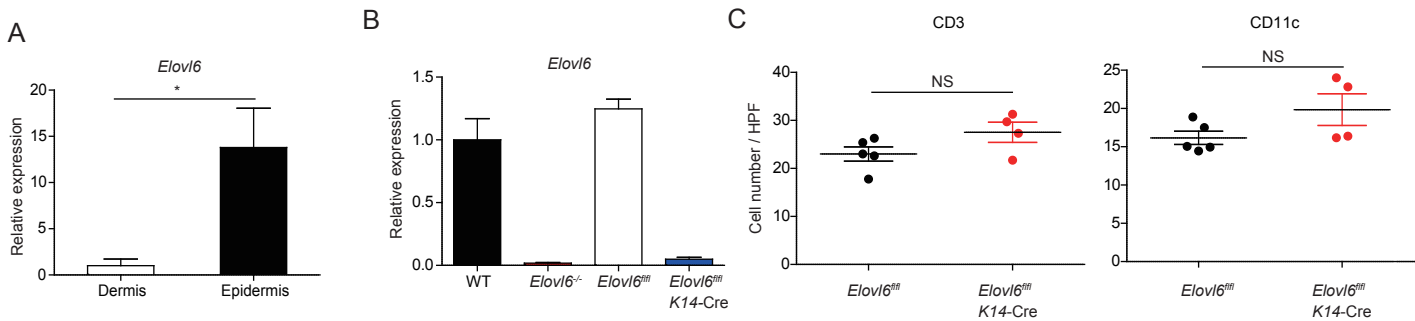

Figure S2

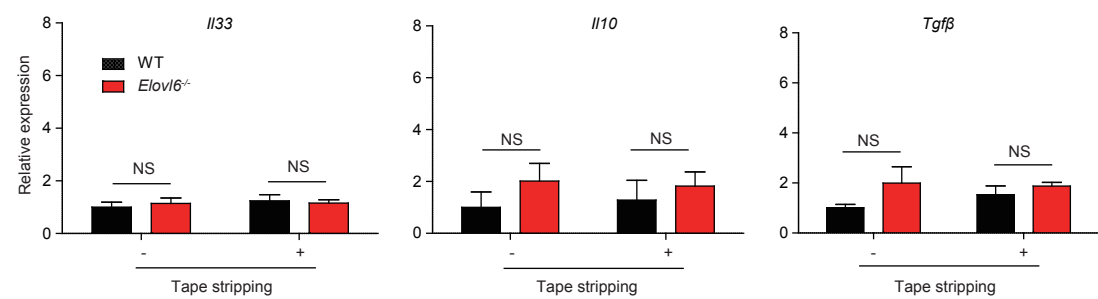

Figure S3

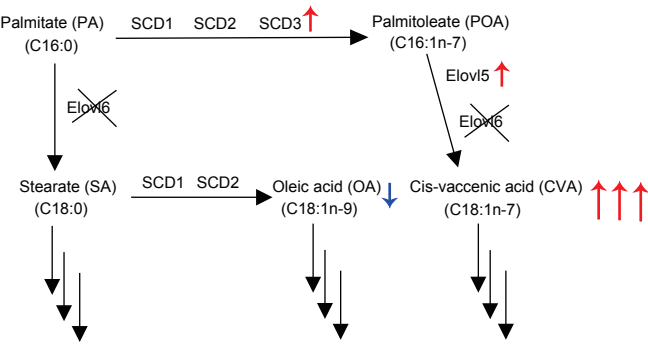

Figure S4

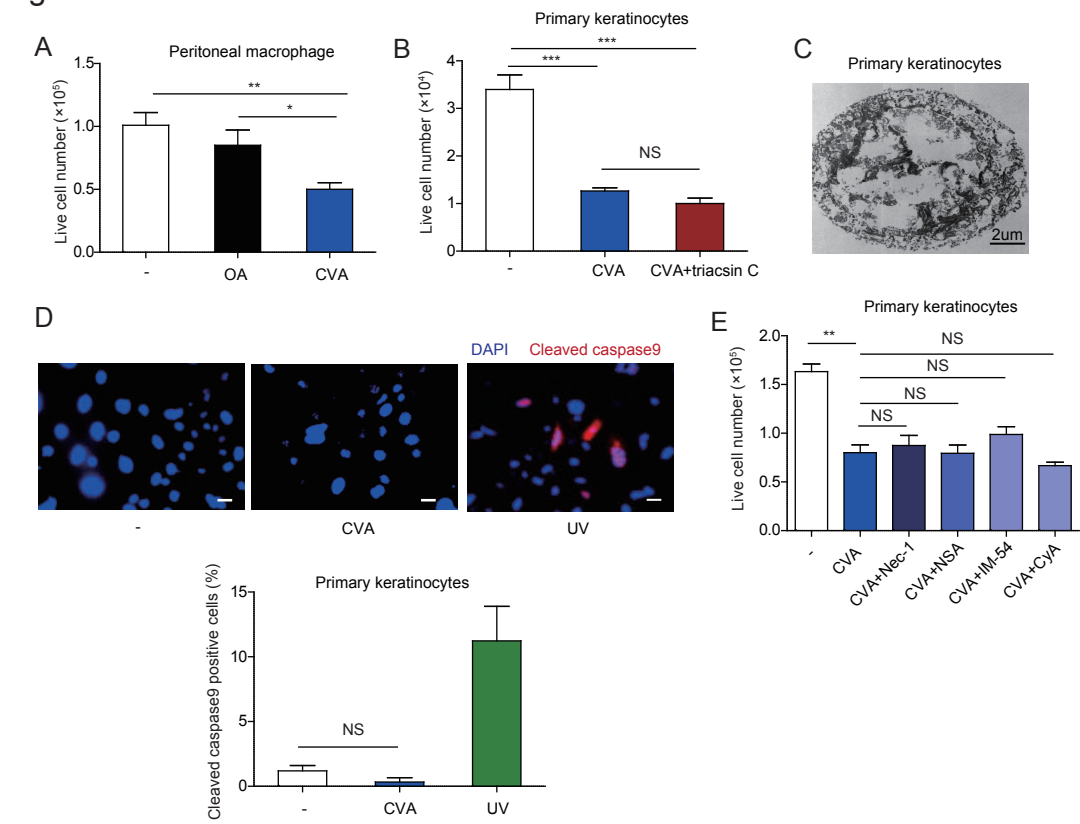

Figure S5

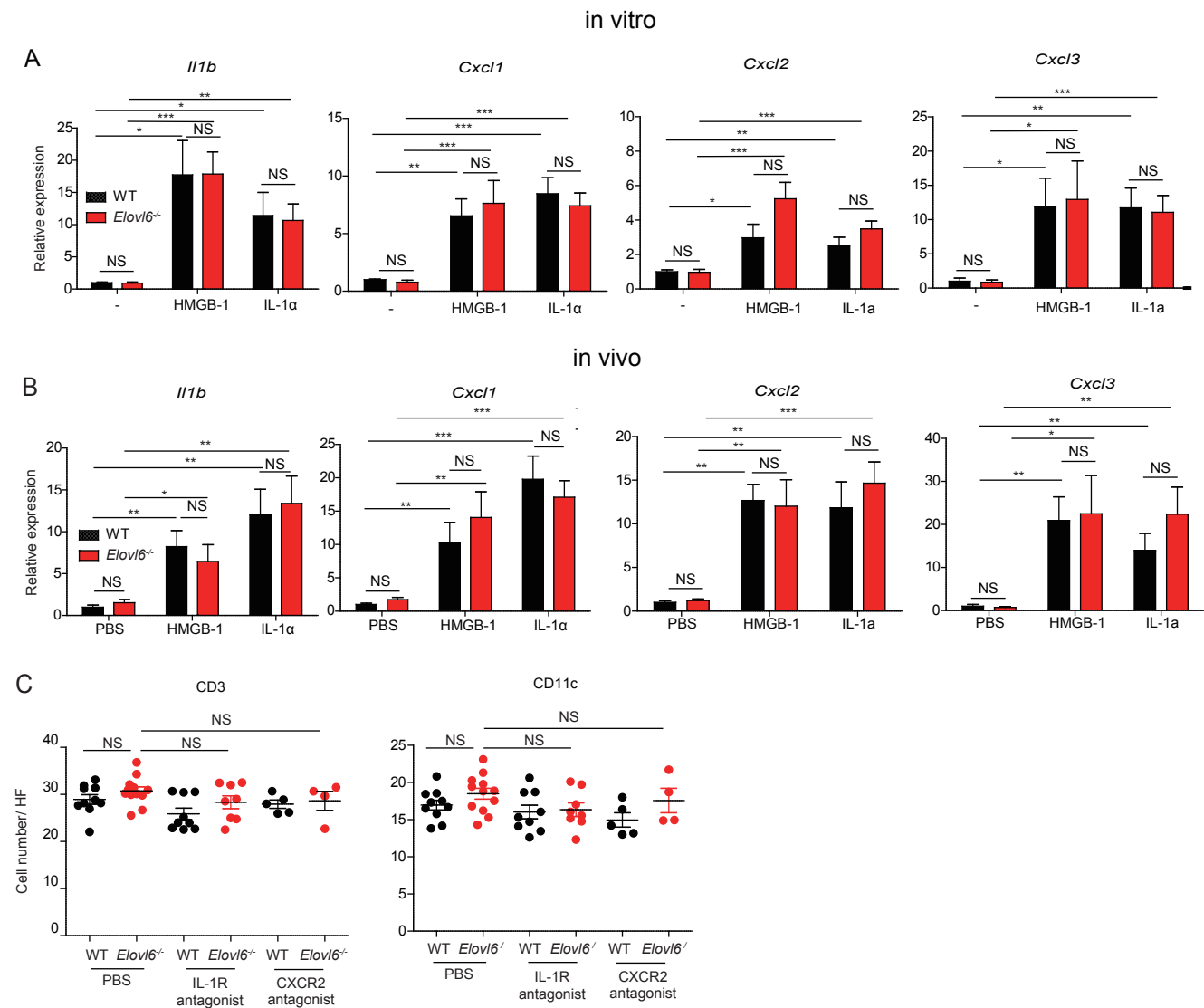

Supplement: Supplementary file 1 — Supplementary figures [file 41419_2018_1226_MOESM1_ESM.pdf]
