# Supplementary material for: Elovl6 regulates mechanical damage-induced keratinocyte death and skin inflammation
Source: Cell Death Dis. 2018 Dec 5;9(12):1181. doi: 10.1038/s41419-018-1226-1 (PMC6281680; doi:10.1038/s41419-018-1226-1)
Supplement: Supplementary file 2 — supplementary figure legends [file 41419_2018_1226_MOESM2_ESM.docx]

**Supplementary Fig. 1**

(A, B) Quantitative RT-PCR analysis of *Elovl6* from dermis and epidermis (n = 3 per group) (A) and from the epidermis of wild-type (WT), *Elovl6^-/-^*, *Elovl6^fl/fl^*, *Elovl6^fl/fl^* *K14*-Cre mice (n=3 to 5 in each group) (B). (C) Numbers of T cells (CD3) and dendritic cells (CD11c) in the skin of *Elovl6^fl/fl^* (n = 5) and *Elovl6*^fl/fl^*K14*-Cre (n = 4) on day 9 after the start of tape stripping. Error bars indicate SD. **P*<0.05. NS, not significant. Data are representative of more than two independent experiments.

**Supplementary Fig. 2**

Quantitative RT-PCR analysis for expressions of cytokines and chemokines in the epidermis of wild-type and *Elovl6^-/-^* mice isolated before and 6 h after tape stripping (n = 10 in each group). NS, not significant. Data are representative of two independent experiments.

**Supplementary Fig. 3**

Schematic representation of the proposed pathway (black arrows) controlling CVA generation in *Elovl6^-/-^* keratinocyte. Red and blue arrows indicate the increase in SCD3, Elovl5, and CVA and the decrease in OA, respectively. CVA, cis-vaccenic acid; OA, oleic acid; SCD3, stearoyl-CoA desaturase 3; Elovl5, elongation of long-chain fatty acids 5.

**Supplementary Fig. 4**

(A) Live cell number of primary peritoneal macrophages 16 h after stimulation with 300 μM of OA or CVA. Live cells were counted using trypan blue exclusion test (n = 3 in each group). (B**,** E) Primary keratinocytes isolated from neonatal skin were cultured for 6 h in the presence or absence of 10 μM of triacsin C (B), or 1 mM necrostatin-1, 1 mM necrosulfonamide (NSA), 2 mM IM-54, or 1 mM cyclosporine A (CyA) (E), followed by stimulation by adding 300 μM CVA; live cells were counted using trypan blue exclusion test 16 h afterward (n = 3). (C) A representative dead primary keratinocyte induced by stimulation with 300 μM CVA for 10 h under a transmission electron microscope. (D) Immunofluorescence microscopic study of primary keratinocyte 10 h after stimulation with 300 μM CVA or 6 h after ultraviolet irradiation. Cells were stained with anti-cleaved caspase 9, followed by Alexa Fluor 594-conjugated secondary antibody and DAPI. White bars indicate a scale (20 μm). Percentage of cleaved caspase 9-positive cells was calculated (n = 3). Error bars indicate SD. *, *P* < 0.05; **, *P* < 0.01, ***, *P* < 0.001; NS, not significant; OA, oleic acid; CVA, cis-vaccenic acid; Nec-1, necrostatin-1; NSA, necrosulfonamide; CyA, cyclosporine A; UV, ultraviolet; DAPI, 4',6-diamidino-2-phenylindole. Data are representative of more than two independent experiments.

**Supplementary Fig. 5**

(A, B) Quantitative RT-PCR analysis of *Il1β*, *Cxcl1*, *Cxcl2* and *Cxcl3* in primary keratinocytes isolated from the neonatal skin of wild-type and *Elovl6^-/-^* mice after stimulation or not with HMGB-1 or IL-1α in vitro (n=10 per group) (A) and in the epidermis isolated 4 h after injection intradermally with PBS, HMGB-1, or IL-1α (n = 8 per each group) (B). (C) Wild-type and *Elovl6^-/-^* mice received intradermal and intraperitoneal administration of PBS (n = 10 and 12, respectively), an IL-1 receptor antagonist (n = 9 and 8, respectively), or a CXCR-2 antagonist (n = 5 and 4, respectively) daily for 9 days, from the beginning on the day of tape stripping. The skin was then analyzed for the number of T cells (CD3) and dendritic cells (CD11C) by immunohistochemical studies. Error bars indicate SD. *, *P* < 0.05; **, *P* < 0.01, ***, *P* < 0.001; NS, not significant. Data are representative of more than two independent experiments.
