# Supplementary material for: Elovl6 regulates mechanical damage-induced keratinocyte death and skin inflammation
Source: Cell Death Dis. 2018 Dec 5;9(12):1181. doi: 10.1038/s41419-018-1226-1 (PMC6281680; doi:10.1038/s41419-018-1226-1)
Supplement: Supplementary file 3 — supplementary experimental procedures [file 41419_2018_1226_MOESM3_ESM.docx]

**Supplementary Experimental Procedures**

**qRT-PCR.** Total RNA was isolated by using ISOGEN (Wako Pure Chemical). Quantitative real-time PCR analysis was performed on a 7500 Fast Real-Time PCR System (Applied Biosystems, Foster City, California, USA) with Power SYBR Green PCR Master Mix (Applied Biosystems). Results are presented relative to those of the housekeeping gene encoding GAPDH (*gapdh*). Primers used were as follows: *Il1b* fwd, GAAATGCCACCTTTTGACAGTG; *Il1b* rev, TGGATGCTCTCATCAGGACAG; *Cxcl1* fwd, CTGGGATTCACCTCAAGAACATC; *Cxcl1* rev, CAGGGTCAAGGCAAGCCTC; *Cxcl2* fwd, AGTGAACTGCGCTGTCAATG; *Cxcl2* rev, GCAAGGCTAACTGACCTGGA; *Cxcl3* fwd, CCCCAGGCTTCAGATAATCA; *Cxcl3* rev, TCTGATTTAGAATGCAGGTCCTT; *Ccl2* fwd, CAGGTCCCTGTCATGCTTC; *Ccl2* rev ATGAGTAGCAGCAGGTGAGTG; *Ccl20* fwd, GGCAGAAGCAAGCAACTACG; *Ccl20* rev, CTTTGGATCAGCGCACACAG; *Tnfa* fwd, CCTGTAGCCCACGTCGTAG; *Tnfa* rev, GGGAGTAGACAAGGTACAACCC; *Il1a* fwd, AGGGAGTCAACTCATTGGCG; *Il1a* rev, TGGCAGAACTGTAGTCTTCGT; *Il6* rev, TCCACGATTTCCCAGAGAAC; *Il10* fwd, GCTGGACAACATACTGCTAACC; *Il10* rev, ATTTCCGATAAGGCTTGGCAA; *Il33* fwd, GGTGAACATGAGTCCCATCA; *Il33* rev, CGTCACCCCTTTGAAGCTC; *Il36a* fwd, GCCTGTTCTGCACAAAGGATG; *Il36a* rev, AGACAGCGATGAACCAACCA; *Il36b* fwd, CACTATGCATGGATCCTCAC; *Il36b* rev, GTCTCTACATGCTATCAAGC; *Il36γ* fwd, ATGGACACCCTACTTTGCTG; *Il36γ*rev, CAGGGTGGTGGTACAAATC; *Tgfb* fwd, tgacgtcactggagttgtacgg; *Tgfb* rev, ggttcatgtcatggatggtgc; *Elovl1* fwd, TCCAAAGCTACCCTCTGATGG; *Elovl1* rev, AGGGAGAGTATCACCAGTGAGA; *Elovl2* fwd, ACGCTGGTCATCCTGTTCTT; *Elovl2* rev, GCCACAATTAAGTGGGCTTT; *Elovl3* fwd, TTCTCACGCGGGTTAAAAATGG; *Elovl3* rev, GAGCAACAGATAGACGACCAC; *Elovl4* fwd, gccctgtggtggtattttgt; *Elovl4* rev, tggtggtacacgtgaaggaa; *Elovl5* fwd, GGTGGCTGTTCTTCCAGATT , *Elovl5* rev, CCCTTCAGGTGGTCTTTCC, *Elovl6* fwd, ACAATGGACCTGTCAGCAAA; *Elovl6* rev, GTACCAGTGCAGGAAGATCAGT; *Elovl7* fwd, CATCGAGGACTGTGCGTTTTT; *Elovl7* rev, CCAGGATGATGGTTTGTGGCA; *Scd1* fwd, TCAACTTCACCACGTTCTTCA; *Scd1* rev, CTCCCGTCTCCAGTTCTCTT; *Scd2* fwd, TGGTTTCCATGGGAGCTG; *Scd2* rev, TTGATGTGCCAGCGGTACT; *Scd3* fwd, CTGACCTGAAAGCCGAGAAG; *Scd3* rev, GCAGAATGCCAGGCTTGTA; *Gapdh* fwd, AGGTCGGTGTGAACGGATTTG; and *Gapdh* rev TGTAGACCATGTAGTTGAGGTCA.

**Analysis of cleaved caspase-9.**

Cells were treated or not with CVA or UV (180 mJ/ cm^2^ for 3 min) and then fixed with 4% paraformaldehyde for 15 min and then with 5 % BSA in PBS containing 0.3% Triton X-100 for 1 h. Subsequently, cells were incubated with rabbit anti-cleaved caspase-9 antibody (1:200; Cell Signaling) for 1 h at room temperature, followed by incubation with Alexa Fluor 594-conjugated donkey anti-rabbit IgG (1:200; Thermo Fisher Scientific) secondary antibody for 30 min. Finally, cells were counterstained with DAPI.

**Cell death analyses**.

To block specific types of cell death or inhibit long-chain acyl-coenzyme A synthetase, cells were pretreated with 1 mM of necrostatin-1 (Cayman Chemicals), 1 mM of necrosulfonamide (Cellagen Technology), 2 mM of IM-54 (Sigma Aldrich), 1 mM of cyclosporine A (Sigma Aldrich), or 10 μM of triacsin C (Abcam) for 6 h before fatty acid stimulation.

**Transmission electron microscopy**. Cultured keratinocytes were fixed by incubating 2.5% glutaraldehyde in PBS (pH 7.4) at 4 °C overnight, postfixed in 1% osmium tetraoxide at 4 °C for 30 min, and then dehydrated through graded concentrations of ethanol. Cells were then transferred to propylene oxide and embedded (Poly/Bed 812, Polysciences, Warrington, Pennsylvania, USA). The samples were analyzed by electron microscopy (JEM-1400, JEOL, Peabody, Massachusetts, USA).
